# Phylogeography in an “oyster” shell provides first insights into the genetic structure of an extinct *Ostrea edulis* population

**DOI:** 10.1038/s41598-021-82020-x

**Published:** 2021-01-27

**Authors:** Sarah Hayer, Dirk Brandis, Alexander Immel, Julian Susat, Montserrat Torres-Oliva, Christine Ewers-Saucedo, Ben Krause-Kyora

**Affiliations:** 1 Zoologisches Museum, Christian-Albrechts-Universität zu Kiel, Hegewischstraße 3, 24105 Kiel, Germany; 2 Institut für klinische Molekularbiologie (IKMB), Christian-Albrechts-Universität zu Kiel, 24118 Kiel, Germany

**Keywords:** Biogeography, Population dynamics, Genetics

## Abstract

The historical phylogeography of *Ostrea edulis* was successfully depicted in its native range for the first time using ancient DNA methods on dry shells from museum collections. This research reconstructed the historical population structure of the European flat oyster across Europe in the 1870s—including the now extinct population in the Wadden Sea. In total, four haplogroups were identified with one haplogroup having a patchy distribution from the North Sea to the Atlantic coast of France. This irregular distribution could be the result of translocations. The other three haplogroups are restricted to narrow geographic ranges, which may indicate adaptation to local environmental conditions or geographical barriers to gene flow. The phylogenetic reconstruction of the four haplogroups suggests the signatures of glacial refugia and postglacial expansion. The comparison with present-day *O. edulis* populations revealed a temporally stable population genetic pattern over the past 150 years despite large-scale translocations. This historical phylogeographic reconstruction was able to discover an autochthonous population in the German and Danish Wadden Sea in the late nineteenth century, where *O. edulis* is extinct today. The genetic distinctiveness of a now-extinct population hints at a connection between the genetic background of *O. edulis* in the Wadden Sea and for its absence until today.

## Introduction

Today, many plant and animal species are threatened or endangered by extinction, mainly due to human influences. The anthropogenically driven species decline exceeds the natural rate^1^. Oysters are prominent examples of endangered species as a result of overexploitation and disease^2,3^. The European flat oyster *Ostrea edulis* Linnaeus, 1758 is native to the shallow regions of the Atlantic coasts from Norway to North East Africa as well as from the Mediterranean Sea to the Black Sea^4–6^. The oyster was very common until the nineteenth century, but the oyster beds eventually declined, at least in part due to overfishing^3^. Subsequently, large translocation efforts were undertaken to sustain the local oyster beds^6,7^. These translocation efforts were unsuccessful in the Wadden Sea, where *O. edulis* went locally extinct in the 1930s^3,7,8^. The decline in the remaining range was further accelerated by introduced pathogens, which led to disease outbreaks in the late 1970s and early 1980s^3,8–11^.

Human activities and their consequences have certainly played a role in the massive decline in wild *O. edulis* populations, but the reasons for the complete extinction of the Wadden Sea population remain poorly understood^8,11^. Present-day phylogeographic studies aimed to create effective resettlement strategies and to support suitable conservation management plans for many species^12–14^. The currently inferred phylogeography of the European flat oyster suggests that populations from the North Sea, the Atlantic and the Mediterranean Sea together with the Black Sea are genetically distinct^12,14^. This pattern matches the general phylogeography of marine species in Europe, which appears to be strongly influenced by the Last Glacial Maximum about 10,000 years ago^15,16^. In many marine species several periglacial refugia have been reconstructed, from where the European coasts were recolonized^16^. The now extinct Wadden Sea population of the European flat oyster could not be included into the phylogeographical studies of present day genetic diversity, which may provide vital clues for the survival of oysters at large.

This highlights a general problem in conservation biology: as soon as a species or population goes extinct, it becomes difficult to reconstruct the reasons for their demise. To remodel the historical phylogeography of extinct populations of *O. edulis*, sequencing historical specimens from museum collections with ancient DNA (aDNA) techniques offers the only possibility to analyse an extinct species or population genetically^17–19^. Only in the last two decades, DNA of mollusc shells has been successfully extracted and sequenced, which encouraged us to analyse a unique museum collection of European oyster shells collected in the 1870s from across Europe with aDNA methods^20–22^.

This study aims to reconstruct the actual state of *O. edulis'* historical phylogeography in its native range between the years 1868 to 1885 by using complete mitochondrial genomes. At this time, the natural oyster beds were still widespread—including the now extinct Wadden Sea. By using the historical shells in combination with the entire genomes, we intend to achieve a higher resolution of the historical phylogeography of *O. edulis* and the reconstruction of the genetic population structure within areas in which *O. edulis* is extinct today.

## Material and methods

### Mitochondrial genome assembly of a present-day oyster

To map historical DNA sequences accurately, we generated a reference mitochondrial genome from a freshly collected individual of *O. edulis*, which was purchased at the Limfjord (Denmark) in October 2018. Modern DNA extraction was performed in the IKMB in Kiel (Germany). 25 mg of soft tissue were extracted with the MagAttract HMW DNAKit (Qiagen) following manufacturers protocol and fragment length was measured using Agilent TapeStation 4200. A Chromium library (10× Genomics)^23^ was prepared and sequenced on one Illumina HiSeq4000 lane. After sequencing, 10× barcodes were removed from reads using Trimmomatic v0.33, specifying HEADCROP:23 for forward reads and HEADCROP:1 for reverse reads^24^. Trimmed reads were used as input for MitoZ with the option “–genetic_code 5” to generate the mitochondrial assembly^25^. The assembly was compared with the existing mitochondrial genome (GenBank acc. no. JF274008) in Geneious v. 2020.0.4^26^. The new mitochondrial genome was uploaded to GenBank with the accession number MT663266.

### Preparation of historical oyster shells

We used museum collection material to generate the historical DNA sequences (see Supplementary Table S1). Shell material stems from the historical oyster collection of the Zoological Museum in Kiel, Germany, which is composed of *O. edulis* shells sampled alive along all coasts of Northern Europe between 1868 and 1885, prior to their decline^27^.

**Figure 1:**
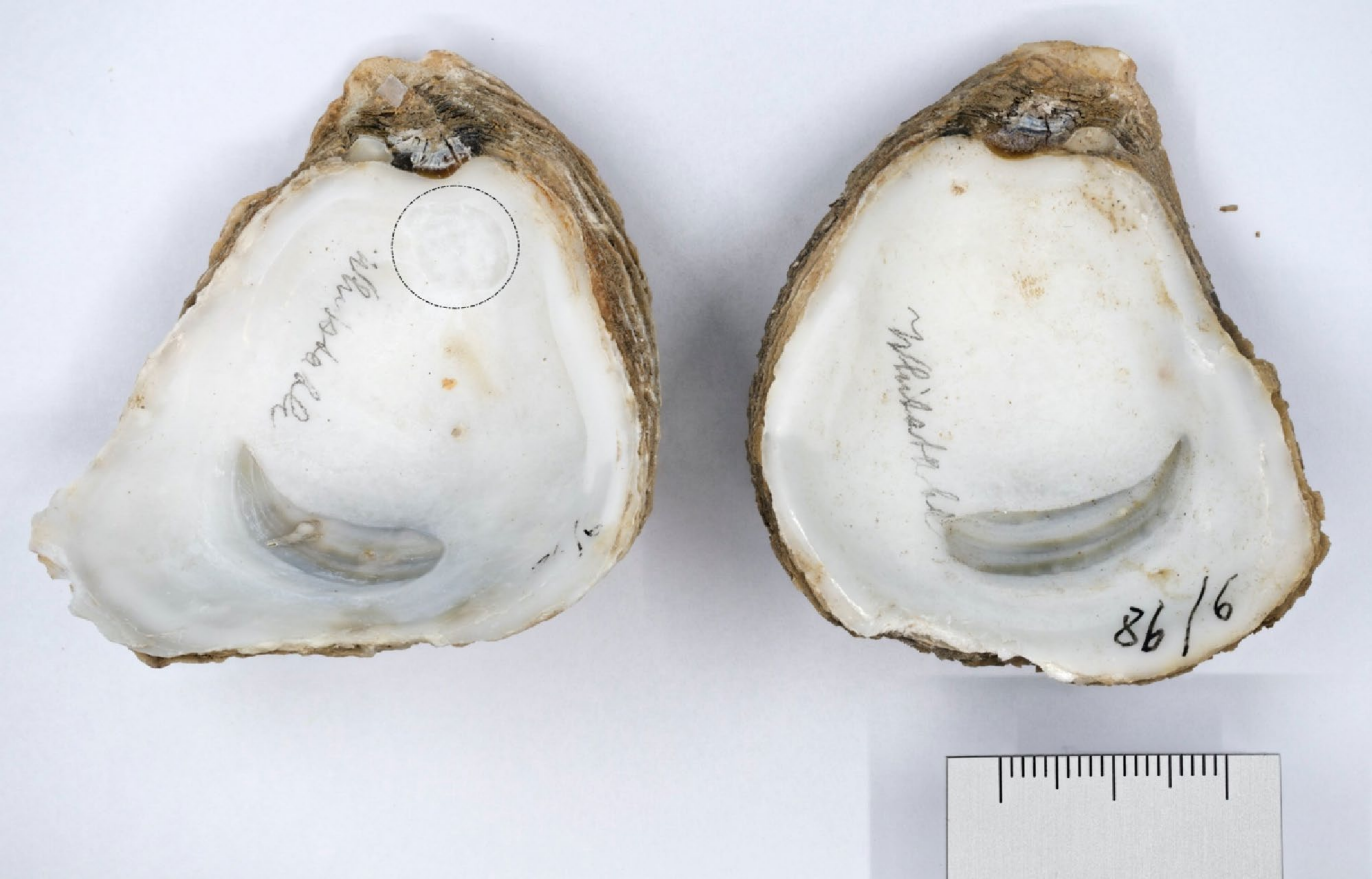
Macroscopic photography of both shells of one individual of *Ostrea edulis,* belonging to the collection of the Zoological museum Kiel. The left shell has a drill hole of approximately 8 mm in diameter underneath the ligament (circled). The right shell is undamaged.

Shells of historical *O. edulis* were bleached, rinsed with distilled water and dried overnight. Using a Dremel hand drill with a round dental drill attachment, the uppermost layer was first removed to exclude any epibiota before a hole was drilled into the inner surface of the shell (see Fig. 1). Generally, the inner side of the shells was used for drilling. Drillings were collected on a weighing paper and transferred into a 2 ml tube. To avoid cross-contamination a new drill bit was used for each sample and the weighing paper was also changed.

### Ancient DNA extraction and sequencing

DNA extraction and pre-PCR steps were performed in clean room facilities dedicated to aDNA research, which were never used for fresh oyster material. DNA extraction of a total of 164 *O. edulis* individuals was performed with approximately 150 mg of shell material following a silica-based protocol with 0.5% SDS added to the extraction buffer to bind the calcium of the shells^20,28,29^. Negative controls for all experimental steps were included to rule out contamination. From each sample, double-stranded DNA sequencing libraries that were partially treated with uracil DNA glycosylase (UDGhalf) were prepared according to an established protocol for multiplex high-throughput sequencing^30^. Sample-specific indices were added to both library adapters via amplification with two index primers. Extraction and library blanks were treated in the same manner. After an initial check for DNA content above 2 ng/µl, only 94 libraries were sequenced on 1/50 of a lane on the Illumina HiSeq 4000 platform (2*75 cycles) in the same Sequencing Centre following the manufacturer's protocol.

### Bioinformatics

De-multiplexing was performed by sorting reads corresponding to their p7 and p5 combinations using the Bcl2fastq software (Illumina, Inc.). All generated sequences were processed according to published protocols specific for aDNA using the EAGER pipeline^31^. We mapped all reads against the newly assembled mitochondrial reference genome of *O. edulis* (GenBank acc. no. MT663266) using the Circular mapper module of the EAGER pipeline with the default setting for aDNA reads ^31,32^. All duplicate reads were removed applying DeDup version 0.12.2, part of the EAGER pipeline, with the default options ^31^. To verify aDNA data sets, we evaluated the presence of postmortem DNA damage signatures from read alignments using mapDamage version 2.0.8^33^. To generate the consensus sequences and a SNP-based multiple alignment, the vcf2genome script was applied. Consensus sequences were built with a coverage of at least 3× against the reference genome, resulting in 65 samples with sequence data. The remaining 29 sequences had a read coverage below three throughout the mitochondrial genome. The read coverage describes the number of reads over a position in the genome.

### Phylogenetic reconstruction

For the phylogenetic reconstruction, we aligned all 65 sequences to our new complete mitochondrial genome sequence of *O. edulis* (see first section). We rooted the phylogenies with two outgroups, *Ostrea lurida* and *Ostrea denselamellosa* (acc. nos. KC768038, NC_015231)^34^ from NCBI GenBank. In order to keep as many sequences as possible, while building a robust phylogeny, we extracted the single nucleotide polymorphisms (SNPs) of each sequence based on its VCF file and calculated the percentage of unidentified SNPs per sequence. Based on this calculation, we reconstructed phylogenies with all sequences that fell below a certain percentage of missing SNPs. We compared phylogenies with more than 95% to more than 60% of missing SNPs in all sequences in 5% steps. This was tested by calculating Neighbour-Joining trees applying the Tamura-Nei Genetic Distance Model and 100 bootstrap replicates in Geneious. The tests resulted in a robust phylogeny based on 37 sequences that had no more than 90% unidentified SNPs. Thus, 28 sequences with more than 90% unidentified SNPs were removed. Detailed information about the bioinformatics of each sequence is given in Supplementary Table S2. The 37 aligned sequences are available through the European Nucleotide Archive (ENA) under the accession number PRJEB40678.

After the initial assessment of the effect of missing data on the phylogenetic reconstruction, we reconstructed a maximum likelihood phylogeny using the General Time Reversible model of complete mitochondrial genomes in MEGA version X^35,36^. We used all sites and estimated the transition/transversion ratio. Branch support was calculated from 500 bootstrap replicates. The branches of all phylogenies were collapsed when the bootstrap support was under 35% using TreeGraph2 version 2.15.0.887^37^. The haplogroups were primarily selected by a number of diagnostic SNPs that are shared by sequences and are specific for a haplogroup. The primarily selection was supported by bootstrap support.

To compare historical and current sequences, we downloaded a total of 16 haplotypes of the 12S rRNA fragment from NCBI GenBank (acc. nos. AY157516 to AY157529, HQ259072, JQ611471)^12,38^. Combining these present-day with our historical 12S rRNA sequences from the mitochondrial genomes, we built a second maximum likelihood phylogeny with the same settings using the shortest 12S rRNA fragment of 280 basepairs as reference. All historical sequences without identified nucleotides within the 12S rRNA encoding gene fragment were automatically removed.

### Population genetic analyses

All population genetic analyses were conducted in R version 3.6.2^39^. Many of the historical sequences contained a high frequency of unmapped nucleotides (“N”s), which prohibit some population genetic analyses. In particular, any site with missing data may be removed from analyses, which would mean the exclusion of most of the variable sites from analyses. To be able to nonetheless conduct population genetic analyses based on sequence similarity, we restricted our analyses to the comparison of haplogroups, not individual haplotypes. For every haplogroup, we extracted the sequence with the highest quality and fewest number of missing nucleotides and multiplied it with the number of individuals present in the respective haplogroup. We are aware that any nucleotide diversity within the haplogroups is lost by this treatment, but since diversity or population size analyses were not the goal of this study, we decided to restrict our analyses to comparisons based on haplogroups.

The genetic differentiation between all population pairs was calculated as Nei’s G_ST_ and Jost’s D with the functions ‘pairwise_G_ST__Nei’ and ‘pairwise_D’ of the ‘mmod’ package^40^. G_ST_ is a derivative of the classical fixation index F_ST_, developed for data with more than two alleles at a locus^41^. We estimated significant deviations from zero (no differentiation between population pairs) by comparison with an empirical distribution of G_ST_ values based on 1000 permutations (Jost’s D calculation in Supplementary Table S4).

## Results

We assembled a new complete mitochondrial genome of *O. edulis* (GenBank acc. no. MT663266) that differs from the existing mitochondrial genome of *O. edulis* in GenBank (acc. no. JF274008) in the COI region (see Supplementary Table S3). It is with 16 356 nucleotides 36 bp longer than the existing mitochondrial genome (JF274008) and encodes 38 genes, including 12 protein-coding genes (PCGs), 3 rRNAs and 23 tRNAs on the same strand (see Supplementary Fig. S1).

The reference genome (JF274008) misses 36 bp in the COI gene, but none of the ancient mitochondrial genomes reveals a deletion in the COI region. We sampled 164 *O. edulis* shells, but only 94 samples contained DNA and were sequenced using Illumina HiSeq. The remaining 57 sequences could not be used for the phylogeny due to low read coverage (minimal coverage for base call ≥ 3). The final alignment is composed of 37 ancient mitochondrial sequences of *O. edulis*, plus both reference genomes and two outgroups (see Supplementary Table S1–S2).

### Phylogenetic reconstruction and population genetic analyses

**Figure 2:**
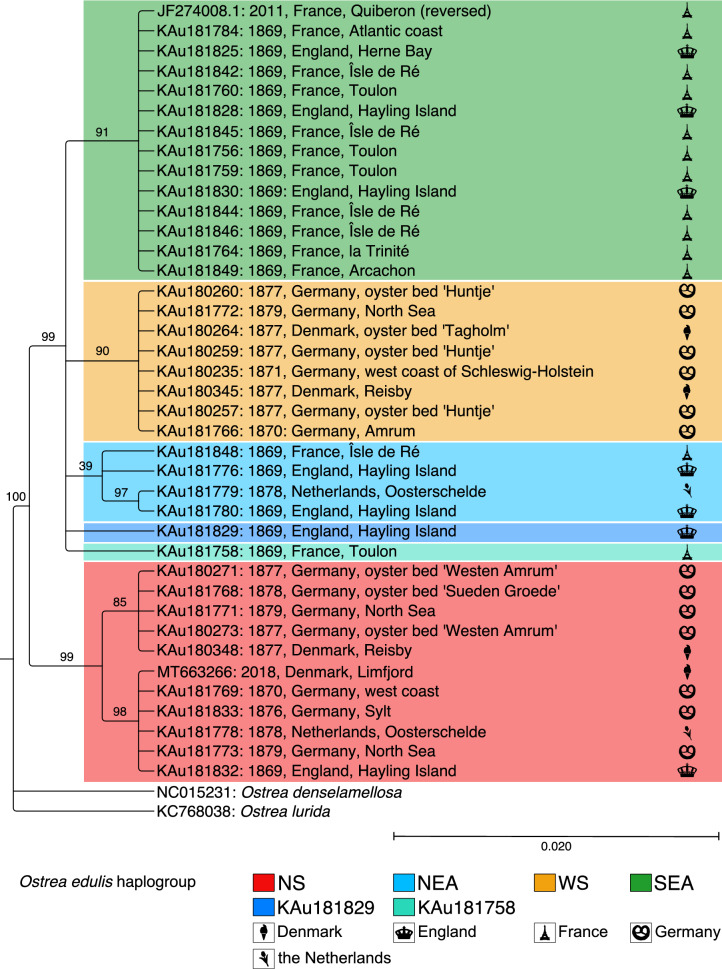
Phylogeny of ancient mitochondrial genomes mapped against MT663266 (complete mitochondrial genome generated in this study) and controlled with JF274008.1 (complete mitochondrial genome in GenBank) using the maximum likelihood method and General time Reversible model using all sites. Bootstrap node support (in percent, from 500 replicates) is shown next to the branches. All branches with less than 35 bootstrap support are collapsed. Colour shading highlights different haplogroups. Phylogeny is rooted with *O. lurida* and *O. denselamellosa*. This analysis involved 41 nucleotide sequences with a total of 16,653 positions in the final dataset. Legend: NS = North Sea, NEA = North East Atlantic, WS = Wadden Sea, SEA = South East Atlantic.

**Figure 3:**
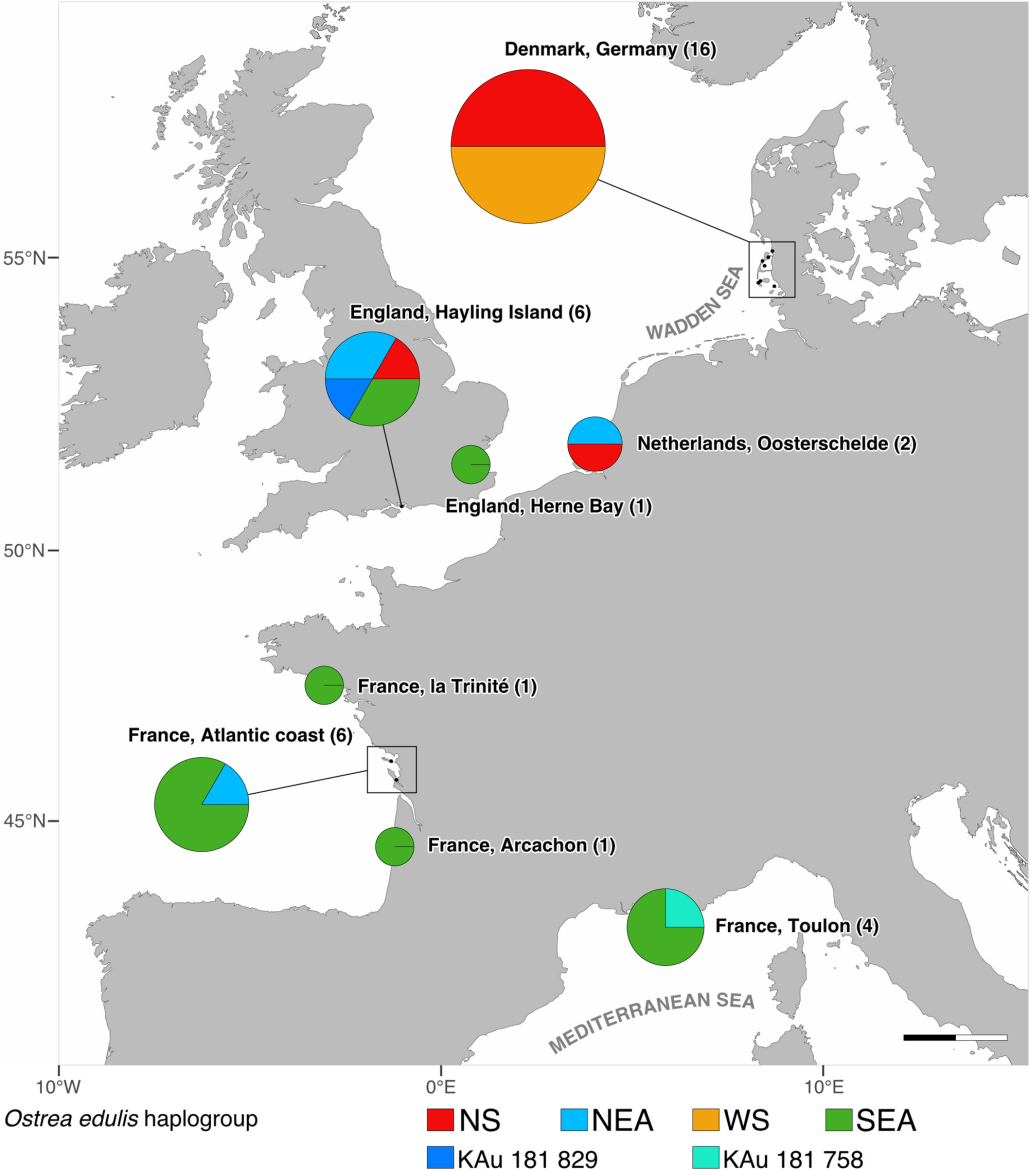
Geographic distribution of mitochondrial haplogroup frequencies of *Ostrea edulis* in Europe between 1869 and 1879. The smallest pie chart represents a single individual. All sample sizes are given in brackets on each location. Scale bar = 100 km. legend: NS = North Sea, NEA = North East Atlantic, WS = Wadden Sea, SEA = South East Atlantic. Map was created using R Studio version 1.1.453 (https://rstudio.com)^39^.

Based on the phylogenetic reconstruction of the complete mitochondrial genome we identified four monophyletic haplogroups, labelled according to their geographic range as Wadden Sea (WS), North Sea (NS), North-East Atlantic (NEA) and South-East Atlantic (SEA) (see Figs. 2, 3). They are defined by a number of diagnostic SNPs that are in this composition unique for a haplogroup (see Supplementary Table S5). Haplogroup WS is based on eight sequences collected only along the Wadden Sea of Germany and Denmark, which is the most restrict distribution range of all haplogroups (Figs. 2, 3). Haplogroup NS is a separate basal clade of eleven sequences that can only be found at the North Sea coasts. It is composed of two branches: One branch is composed of more local sequences that were collected at the North Frisian and Danish Wadden Sea islands and circumjacent oyster beds. The second branch includes sequences from all coasts of the North Sea like the Netherlands, England and Germany. Our new reference also falls into that branch (Figs. 2, 3). Haplogroup SEA is composed of 13 individuals collected at the Atlantic and Mediterranean coasts of France and the South coast of England in 1869 (Figs. 2, 3). The mitochondrial genome from GenBank, which originates from an oyster collected at the French Atlantic coast, also belongs to this haplogroup. Haplogroup NEA holds four aDNA sequences from the coasts of France, the Netherlands and England between 1869 and 1878 (Figs. 2, 3). This haplogroup is composed of sequences that have many variable SNPs, and given more data, we might have split this haplogroup up in two haplogroups. Two sequences from Hayling Island, England and Toulon, France were not assigned to any haplogroup.

In comparison to the phylogeny based on the complete mitochondrial genome (Fig. 2), the haplogroups could not be resolved in the 12S rRNA phylogeny (see Supplementary Fig. S2). The phylogeny is also poorly supported by bootstrap values, which makes it difficult to draw conclusions about a relationship between aDNA samples and formerly defined haplotypes^12,38^.

In order to identify the genetic differentiation between populations, calculations of Nei’s G_ST_ were conducted between populations with four or more individuals and ranged from 0 to 0.343 (Table 1). Since the number of individuals per sampling site was sometimes only one or two, we combined several of the sampling locations: all specimens from England were summarized, as were the individuals collected along the French Atlantic coast, and German and Danish individuals were condensed to one population called “Wadden Sea”. The Netherlands were excluded from the statistical calculations, since the number of individuals was very small. The model revealed partly a significant population structure across Europe (Table 1). The Wadden Sea is most distinct from the rest of Europe (Table 1). The populations from England are similar to those of the Mediterranean and the Atlantic coast of France, but distinct from the Wadden Sea (Table 1). The Atlantic and Mediterranean coasts of France are similar to each other, given that most samples can be assigned to haplogroup SEA (see Table 1).

**Table 1:** Results of Nei’s G_ST_ pairwise analyses of *O. edulis* populations from sampling sites with more than four sampled individuals. Pairwise p-values are shown in the top triangle (italics background). Values with significance are bolded (p-value < 0.05).

	Wadden Sea	England	Atlantic	Mediterranean
Sample size	20	7	8	4
Haplogroups	NS, WS	NS, NEA, Kau181829, SEA	NEA, SEA	Kau181758, SEA
**Nei’s G**_**ST**_				
Wadden Sea	–	***0.043***	***0.004***	***0.021***
England	**0.1367843s0**	–	*0.163*	*0.473*
Atlantic	**0.34252229**	0.06331849	–	*0.472*
Mediterranean	**0.31563499**	0.04836342	0.02782109	–

## Discussion

In the course of this study, we reconstructed 37 complete historical mitochondrial genomes extracted from dry shells of *O. edulis* and based on this aDNA data, we successfully reconstructed the phylogeography of *O. edulis* throughout the native range from the 1870s. The phylogeographic reconstruction revealed the presence of an autochthonous population in the German and Danish Wadden Sea, where the European oyster is extinct today. This finding provides a possible reason for the extinction of the European oyster in the Wadden Sea and for its continuous absence until today.

The most astonishing result is the autochthonous haplogroup WS, which is based on eight individuals that were without exception collected from the near shore of the Danish and German Wadden Sea, where the European oyster went extinct in the 1930s^8^. Its restriction to the Wadden Sea may indicate local adaptation to the distinct environmental conditions of the Wadden Sea. The oyster beds in the Wadden Sea were situated in deeper trenches between the mudflats, so that they were still covered with water during low tide^27^. But due to the tidal change, currents and salinity in the deeper trenches are more extreme than in the central North Sea or the Atlantic coast. While haplogroup WS may have been well-adapted to the extreme environmental conditions of the Wadden Sea, it may not have been able to react to broader ecological changes or might have been particularly vulnerable to diseases, high temperatures, pollution or other global change threats. This possible vulnerability to ecological changes in combination with overfishing could have led to the extinction event of haplogroup WS in the early twentieth century. This would also explain why *O. edulis* has not recovered in the Wadden Sea.

However, this does not explain why the second haplogroup NS, which was also present in the Wadden Sea in the late nineteenth century, has not re-occupied this region. Especially since this haplogroup survived in the adjacent Limfjord. One explanation could be the different environmental conditions between the Wadden Sea and the Limfjord: The Limfjord is a shallow water body of 7 m in average and opens through the Thyborøn canal into the North Sea, which is a canal of 1 km in width. This narrow connection to the North Sea leads to a low volume inflow of salt water into the Limfjord, which results in a lower salinity towards the East and modified tidal ranges and currents. Due to these environmental conditions, the Limfjord is less disturbed and thus a safer location for oysters to grow. Because of the remote location combined with reduced water inflow and currents, pathogens and competitors like *Bonamia ostreae* and *Magallana gigas* appeared in the Limfjord later than at other locations in Europe^42,43^. Another hypothesis is based on the results of Ronza et al.^44^: they showed that *O. edulis* has not developed any resistance to bonamiosis in the Limfjord yet. The presence of *B. ostreae* in the North Sea may therefore be a reason why the native oyster has not recolonized the Wadden Sea again^44^. In the past, it has been observed that *B. ostreae* was eliminated during cold winters, while *O. edulis* was able to survive^45^. However, such strong winters have become scarce in the course of the global warming, which may make it difficult for *O. edulis* to re-occupy the Wadden Sea, when climatic conditions favour the survival of bonamiosis. Hence, the environmental difference between the remote Limfjord and the Wadden Sea, altered climate conditions and parasites could explain why *O. edulis* has not resettled the Wadden Sea again.

Moreover, the Limfjordian oyster beds are a special case concerning the survival of natural beds of the European flat oyster, since it is the only surviving natural oyster bed in the North Sea. The Limfjord oyster beds are young in comparison to the oyster beds along the North Sea coast. Initially a brackish water body, the Limfjord became marine when a heavy storm connected it to the North Sea in 1825^6^. Soon after, the first oyster beds were discovered in the Limfjord^6^. The Limfjordian oyster beds were, however, also supported by translocations of 18 million adult oysters originating from the Netherlands^7^. Our genetic results only verify the survival of haplogroup NS in the Limfjord, but cannot differentiate if this haplogroup came into the Limfjord via natural dispersal or translocation.

Another conspicuous result concerns our phylogenetic reconstruction revealing a split between the most basal haplogroup NS and the remaining lineages. This relatively old split could have evolved when oysters were constrained in their distribution to two different glacial refugia. The basal haplogroup NS is widely spread in the central North Sea with the most western record in the English Channel, but no individuals with this haplotype were retrieved further south (see Fig. 3). It represents therefore a private haplogroup to the North Sea, which is indicative of a northern glacial refugium^16^. The Hurd Deep, a deeper trench in the western English Channel, and some ice free coasts along the North Sea could have been glacial refugia to different seaweed species^46–49^, molluscs^50,51^ and decapods^52^. Curiously, this northern lineage has not expanded further south after the glaciers retracted. Larval dispersal may be constrained by the internal counter clockwise circulation currents in the North Sea^53^. It would also be conceivable that this northern lineage is not well-adapted to southern environmental conditions such as high temperatures. Of course, it is not possible to draw conclusions about adaptation processes based on the results of the mitochondrial genome, but other phylogeographic studies also show that the distribution of genetic lineages correlates with environmental conditions^54^.

Nevertheless, this study could prove the presence of haplogroup NS in the Wadden Sea at the end of the nineteenth century and assuming that this haplogroup is specially adapted to this environment, this provides a valuable result for conservation management and resettlement strategies of the European flat oyster in the Wadden Sea.

The southern clade presents with three haplogroups (WS, SEA & NEA) a higher phylogenetic diversity than the northern clade. It is distributed along the East Atlantic coast and the Mediterranean Sea. We propose that this clade had one or more southern glacial refugia, and expanded northwards as the climate warmed. The presence of several monophyletic groupings within this clade could be due to differentiation processes during its northward spread. The northwards expansion could have led to a series of founder effects presenting the different haplogroups restricted to a geographic range such as haplogroup WS. This recolonization of Northern Europe from southern glacial refugia is common in both marine and terrestrial species^47,55–60^.

Based on our data, the Atlantic and Mediterranean populations are not significantly different from each other, as might have been expected given the biogeographic break between them (see haplogroup SEA, Fig. 3). Phylogeographic studies consistently separate the Atlantic from the Mediterranean Sea not only in the European oyster^12^, but also in a diverse array of marine organisms such as marine algae and seagrasses^46,48,61^, different invertebrates^62–65^, bony fishes^66–69^ and cartilaginous fishes^70^. Due to the low sample size of *O. edulis* in the Mediterranean Sea—we were only able to sequence four individuals successfully, the missing genetic differentiation between the historical Atlantic and Mediterranean populations of *O. edulis* is likely an artefact. Moreover, all sequenced individuals were collected from a single location in the Mediterranean Sea.

At the end of the nineteenth century, the anthropogenic influence on the oyster beds by exchanging oyster spat all over Europe was already common to sustain the oyster beds^3,6,7^. However, the haplogroups in our study do not reflect these massive translocations and are restricted to geographical ranges with the exception of haplogroup NEA. The patchy distribution of this haplogroup (see Fig. 3) could be the result of translocations. Fullarton stated that by the 1800s “most of the English natives are born in France or Holland, and are fattened at Whitstable or other beds in the South of England”^71^. Bromley and colleagues pictured that imports to England originated from France, Scotland, the Netherlands, Ireland and other unknown source countries^7^. However, we could not include any oysters from the French coast of the English Channel or other locations of Great Britain or Ireland to further confirm these hypotheses. Another explanation would be that this haplogroup does not actually represent a cluster of highly similarly sequences. Haplogroup NEA has the lowest bootstrap support (39%, see Fig. 2) and contains somewhat disparate sequences. Again, the inclusion of more sequences could resolve this potential artefact.

Present-day phylogeographical studies identified both significant differentiation between the Atlantic and Mediterranean and a differentiated clade in the North Sea^12,14,72^. This study on the complete mitochondrial genome confirms the phylogeographical pattern of *O. edulis,* however, the resolution is much higher and finer due to more SNPs and more sampling sites, which still existed in the late nineteenth century in comparison to today. Since all four studies found the same phylogeographic pattern, we conclude that this seems to be a temporally stable pattern over the past 150 years despite large-scale translocations. Additionally, our reference mitochondrial genome originating from the Limfjord (Denmark) in 2018 confirms that haplogroup NS still exists today (see Fig. 2).

Danic-Tchaleu et al.^32^ also assembled a mitochondrial genome of *O. edulis*, which originates from Quiberon (France). They used long range PCR and Sanger sequencing^32^. Since we used high-throughput sequencing for the Limfjordian oyster, these methodological differences could explain the additional 36 bp in our genome. However, it is significant that not only the new modern mitochondrial genome has 36 bp more, but the 37 historical mitochondrial genomes as well. Therefore, a sequencing error is unlikely.

## Conclusion

This study’s goal was to reconstruct the actual state of the locally extinct *O. edulis'* historical phylogeography at the end of the nineteenth century, at a time when natural oyster beds were still widespread – including the now extinct Wadden Sea population. The reconstruction of the phylogeography of the European flat oyster has reached an important milestone because of two factors: Firstly, the application of complete mitochondrial genomes can resolve the phylogeographic pattern in a more detailed manner than single DNA markers. Secondly, the extraction of DNA from historical individuals housed in natural history collections allows the representation of the exact phylogeography at a specific point in time in the past. As a result, an autochthonous haplogroup could be detected in the Wadden Sea. This finding underlines the importance of interdisciplinary approaches. Both museum collection documentation, which has been proven to be valuable in earlier works on *O. edulis* (Hayer et al. 2019), as well as innovative aDNA methods enable us to gain new results from historical material.

## Supplementary Information

Supplementary 1.
