# Supplementary material for: Phylogeography in an “oyster” shell provides first insights into the genetic structure of an extinct Ostrea edulis population
Source: Sci Rep. 2021 Jan 27;11:2307. doi: 10.1038/s41598-021-82020-x (PMC7840910; doi:10.1038/s41598-021-82020-x)
Supplement: Supplementary file 1 — Supplementary 1. [file 41598_2021_82020_MOESM1_ESM.pdf]

Supplementary Material

Phylogeography in an "oyster" shell - first insights into the genetic structure of an extinct *Ostrea edulis* population

Sarah Hayer[1]*, Dirk Brandis[1], Alexander Immel[2], Julian Susat[2], Montserrat Torres-Oliva[2], Christine Ewers-Saucedo[1] and Ben Krause-Kyora[2]

[1] Zoologisches Museum, Christian-Albrechts-Universität zu Kiel, Hegewischstraße 3, 24105 Kiel, Germany

[2] Institut für Klinische Molekularbiologie (IKMB), Christian-Albrechts-Universität zu Kiel, 24118 Kiel, Germany

* corresponding author

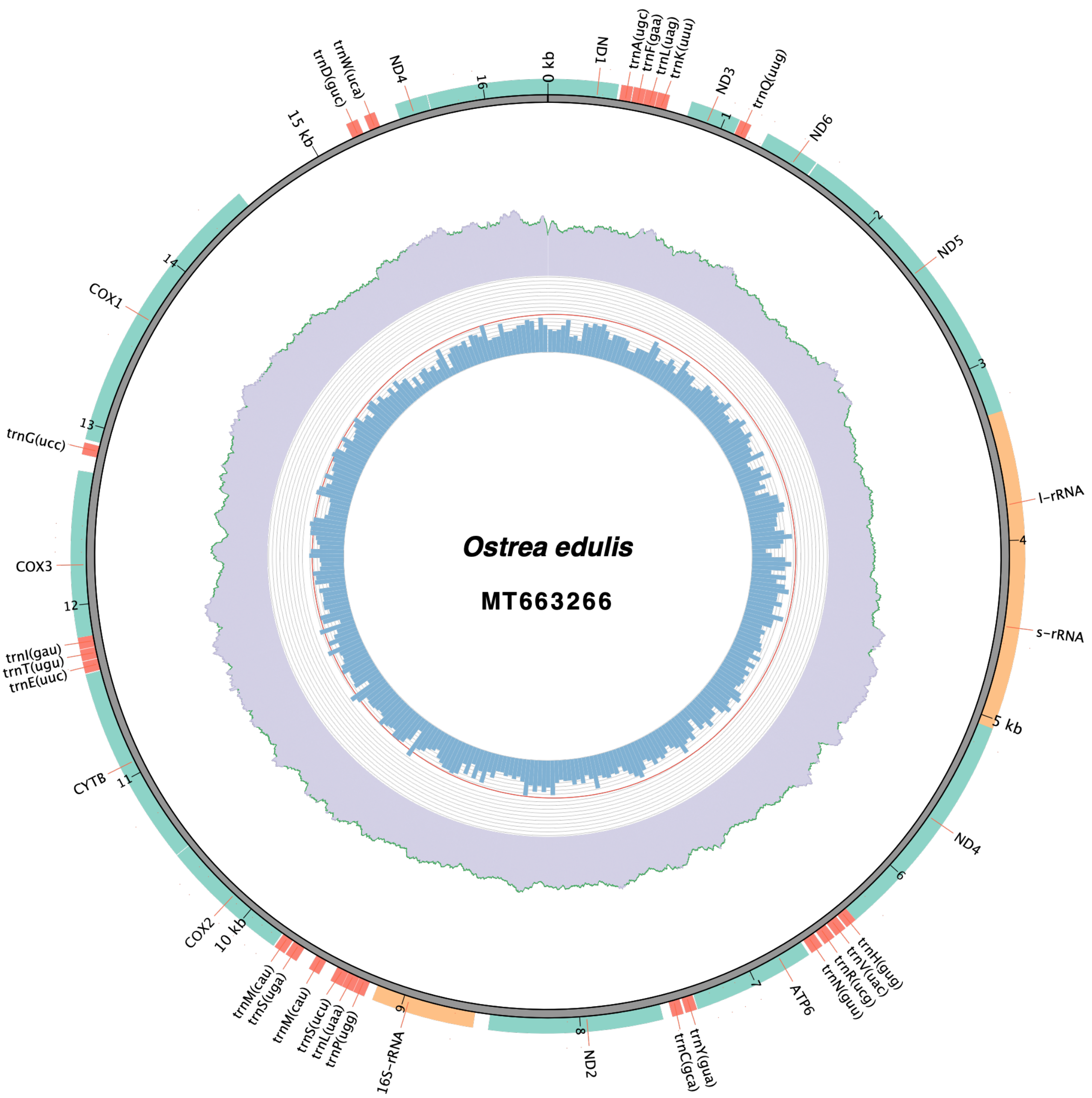

Figure S1. Mitochondrial genome map of *Ostrea edulis* drawn with MitoZ. The innermost circle shows the GC content, the middle circle reveals the depth distribution with a green outline when the depth is larger than the upper quartile. The outermost circle shows the gene features for proteins (green) and rRNAs (yellow) that are abbreviated with standard abbreviations. The one letter amino acid code is used for tRNA (red) designation.

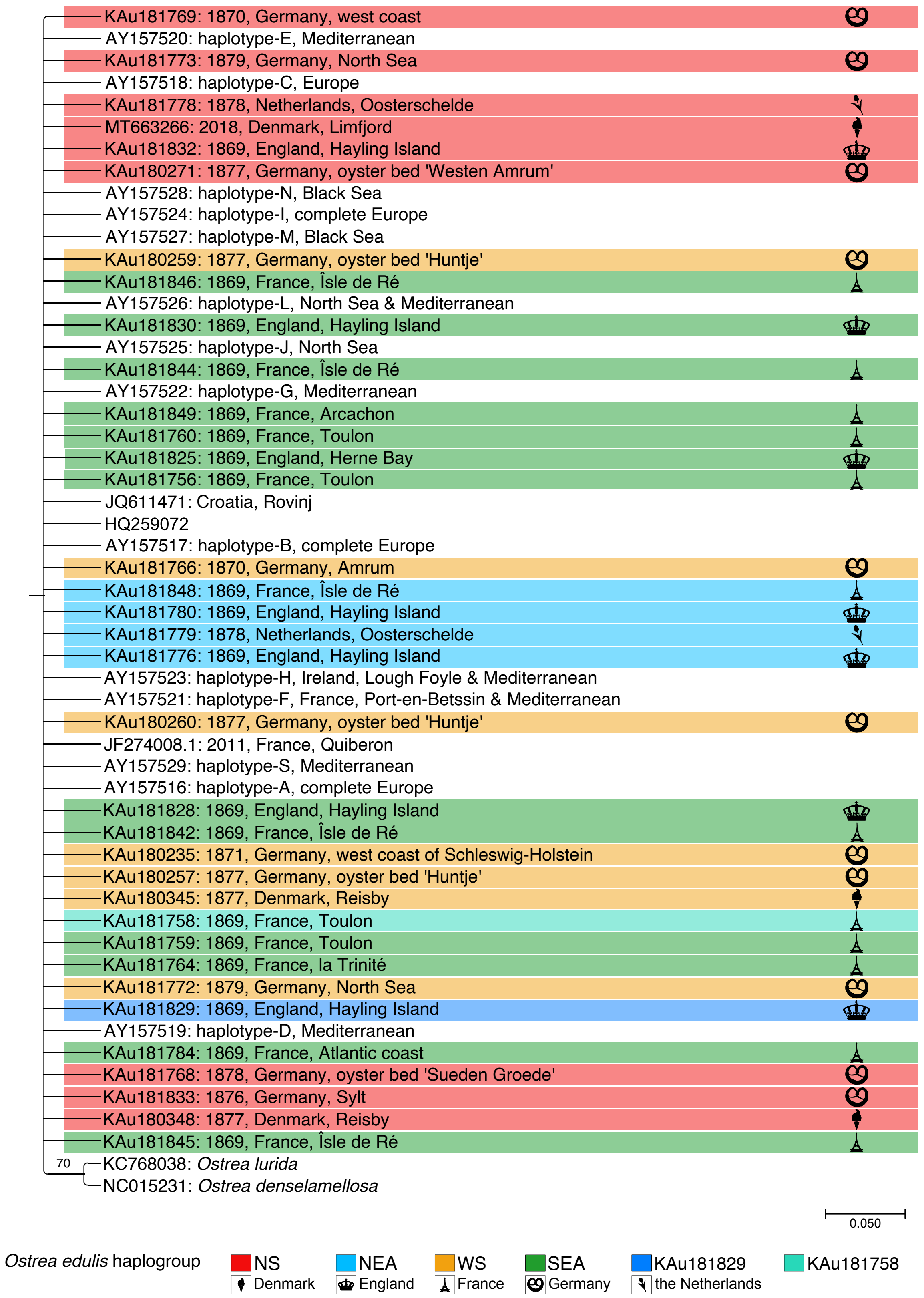

Figure S2. Phylogeny of ancient and modern 12S rRNA sequences mapped against MT663266 (complete mitochondrial genome generated in this study) and controlled with JF274008.1 (complete mitochondrial genome in GenBank) using the maximum likelihood method and General time Reversible model using all sites. Modern DNA was downloaded from GenBank (Diaz-Almela et al., 2004; Malkowsky & Klussmann-Kolb, 2012). Bootstrap node support (in percent, from 500 replicates) is shown next to the branches. All branches with less than 35 bootstrap support are collapsed. Colour shading highlights different haplogroups. Phylogeny is rooted with *O. lurida* and *O. denselamellosa*. This analysis involved 54 nucleotide

sequences with a total of 280 positions in the final dataset. legend: NS = North Sea, NEA = North East Atlantic, WS = Wadden Sea, SEA = South East Atlantic

Table S1. Details of the museum collection material we used to generate the historical DNA sequences. The sequences can be accessed via the European Nucleotide Archive (ENA) under the accession number PRJEB40678.

ID	Museum collection ID	Collection Date	Organism	Sample type	sampling site
KAu180235	Mo 25/1	24.03.1871	*Ostrea edulis*	shell	Germany, west coast of Schleswig-Holstein
KAu180257	Mo 58/2	23.05.1877	*Ostrea edulis*	shell	Germany, Sylt, oyster bed 'Huntje'
KAu180259	Mo 58/4	23.05.1877	*Ostrea edulis*	shell	Germany, Sylt, oyster bed 'Huntje'
KAu180260	Mo 58/5	23.05.1877	*Ostrea edulis*	shell	Germany, Sylt, oyster bed 'Huntje'
KAu180264	Mo 56/2	24.05.1877	*Ostrea edulis*	shell	Denmark, West coast (east coast of Rømø Island), oyster bed 'Tagholm'
KAu180271	Mo 73/5	22.05.1877	*Ostrea edulis*	shell	Germany, west coast of Schleswig-Holstein, oyster bed 'Westen Amrum'
KAu180273	Mo 73/7	22.05.1877	*Ostrea edulis*	shell	Germany, west coast of Schleswig-Holstein, oyster bed 'Westen Amrum'
KAu180345	Mo 47/1 rechts	25.05.1877	*Ostrea edulis*	shell	Denmark, west coast, Reisby-Steert
KAu180348	Mo 47/4 links	25.05.1877	*Ostrea edulis*	shell	Denmark, west coast, Reisby-Steert
KAu181756	Mo 106/7	1869	*Ostrea edulis*	shell	France, Mediterranean coast, Toulon, oyster bed 'la Seyne'
KAu181758	Mo 106/8	1869	*Ostrea edulis*	shell	France, Mediterranean coast, Toulon, oyster bed 'la Seyne'

KAu181759	Mo 106/6	1869	*Ostrea edulis*	shell	France, Mediterranean coast, Toulon, oyster bed 'la Seyne'
KAu181760	Mo 106/4	1869	*Ostrea edulis*	shell	France, Mediterranean coast, Toulon, oyster bed 'la Seyne'
KAu181764	Mo 99/3 rechts	1869	*Ostrea edulis*	shell	France, Atlantic coast, la Trinité
KAu181766	Mo 72/1 rechts	1870	*Ostrea edulis*	shell	Germany, west coast of Schleswig-Holstein, Amrum, 6 years old
KAu181768	Mo 75/1 rechts	1878	*Ostrea edulis*	shell	Germany, west coast of Schleswig-Holstein, oyster bed 'Süden Gröde'
KAu181769	Mo 39/1 rechts	1870	*Ostrea edulis*	shell	Germany, west coast of Schleswig-Holstein
KAu181771	Mo 7/2 links	1879	*Ostrea edulis*	shell	Germany, oyster beds of Schleswig-Holstein, infested by polychaetes
KAu181772	Mo 7/3 rechts	1879	*Ostrea edulis*	shell	Germany, oyster beds of Schleswig-Holstein, infested by polychaetes
KAu181773	Mo 7/4 rechts	1879	*Ostrea edulis*	shell	Germany, oyster beds of Schleswig-Holstein, infested by polychaetes
KAu181776	Mo 90/1 rechts	1869	*Ostrea edulis*	shell	England, English Channel, Hayling Island, near Portsmouth, Hampshire
KAu181778	Mo 80/2 rechts	1878	*Ostrea edulis*	shell	the Netherlands, Oosterschelde, market oysters
KAu181779	Mo 80/3 links	1878	*Ostrea edulis*	shell	the Netherlands, Oosterschelde, market oysters
KAu181780	Mo 92/1 links	1869	*Ostrea edulis*	shell	England, English Channel, Hayling Island (near Portsmouth, Hampshire), 2 years old
KAu181784	Mo 98/1 rechts	1869	*Ostrea edulis*	shell	France, Atlantic coast, la Tremblade (Seudre estuary)

KAu181825	Mo 88/1 rechts	1869	*Ostrea edulis*	shell	England, North Sea coast, Thames estuary, Herne Bay
KAu181828	Mo 91/3 rechts	1869	*Ostrea edulis*	shell	England, English Channel, Hayling Island (near Portsmouth, Hampshire)
KAu181829	Mo 91/5 links	1869	*Ostrea edulis*	shell	England, English Channel, Hayling Island (near Portsmouth, Hampshire)
KAu181830	Mo 91/6 links	1869	*Ostrea edulis*	shell	England, English Channel, Hayling Island (near Portsmouth, Hampshire)
KAu181832	Mo 91/8 rechts	1869	*Ostrea edulis*	shell	England, English Channel, Hayling Island (near Portsmouth, Hampshire)
KAu181833	Mo 51/2 rechts	1876	*Ostrea edulis*	shell	Germany, west coast of Schleswig-Holstein, oyster beds of Sylt, 'with parasites'
KAu181842	Mo 97/2 links	1869	*Ostrea edulis*	shell	France, Atlantic coast, Îsle de Ré
KAu181844	Mo 97/4 rechts	1869	*Ostrea edulis*	shell	France, Atlantic coast, Îsle de Ré
KAu181845	Mo 97/5 rechts	1869	*Ostrea edulis*	shell	France, Atlantic coast, Îsle de Ré
KAu181846	Mo 97/6 rechts	1869	*Ostrea edulis*	shell	France, Atlantic coast, Îsle de Ré
KAu181848	Mo 97/8 rechts	1869	*Ostrea edulis*	shell	France, Atlantic coast, Îsle de Ré
KAu181849	Mo 102/1 rechts	1869	*Ostrea edulis*	shell	France, Atlantic coast, Bassin d'Arcachon (Dép. Gironde)

Table S2. Sequencing statistics of the 37 historical samples of *O. edulis* used for this study.

Sample ID	mapped reads after duplicate removal	average read length	read depth	average coverage
KAu180235	6974	88,48	603587	36,903093
KAu180257	7244	70,87	502730	30,736732
KAu180259	619	90,42	54872	3,354854
KAu180260	520	67,15	34295	2,096784
KAu180264	363	65,82	23485	1,435864
KAu180271	363	79,26	28020	1,713132
KAu180273	240	90,42	21335	1,304414
KAu180345	109884	90,26	9756220	596,491807
KAu180348	240	82,17	19384	1,18513
KAu181756	1693	71,59	119045	7,278368
KAu181758	2680	77,64	203909	12,466923
KAu181759	60645	64,21	3823973	233,796343

KAu181760	3393	70,04	233325	14,265407
KAu181764	4553	86,73	386522	23,631817
KAu181766	15182	65,7	978490	59,824529
KAu181768	431	77,26	32627	1,994803
KAu181769	25378	88,93	2210995	135,179444
KAu181771	275	80,24	21619	1,321777
KAu181772	6453	85	537481	32,861396
KAu181773	5512	74,88	404405	24,725177
KAu181776	7281	71,11	507080	31,00269
KAu181778	2320	71,18	162251	9,919968
KAu181779	2589	79,28	200484	12,25752
KAu181780	785	73,03	56195	3,435742
KAu181784	2495	79,87	195051	11,925348
KAu181825	18165	52,97	941011	57,533076
KAu181828	20610	74,4	1501418	91,79616

KAu181829	537	64,44	33964	2,076546
KAu181830	3589	68,07	239129	14,620261
KAu181832	3059	84,77	253909	15,523905
KAu181833	2140	80,48	168912	10,327219
KAu181842	1820	83,9	149695	9,152298
KAu181844	589	75,48	43664	2,669601
KAu181845	1941	75,45	143543	8,776167
KAu181846	468	71,55	32986	2,016752
KAu181848	23150	71,71	1628234	99,549645
KAu181849	480	77,86	36756	2,247248

Table S3. Comparison of the mitochondrial genomes of *O. edulis* in total and in the COI region.

	JF274008 / MT663266	
	total	COI
Number of nucleotides	16323/16356	1566/1596
Nucleotide difference [%]	0.22	1.9
Number of SNPs	385	81
SNPs [%]	2.4	5.1

Table S4. Results of Jost's D pairwise analyses of *O. edulis* populations from sampling sites with more than four sampled individuals. Pairwise p-values are shown in the top triangle (grey background). Values with significance are bolded (p-value < 0.05).

	Wadden Sea	England	Atlantic	Mediterranean
Sample size	20	7	8	4
Haplogroups	A, B, C, D, E	A, B, D, E	B, E	D, E
**Jost's D**				
Wadden Sea	-	**0.047**	**0.019**	**0.030**
England	**0.166158873**	-	0.316	0.548
France Atlantic	**0.296833074**	0.028293051	-	0.613
Mediterranean	**0.283629472**	0.023071138	0.003467137	-

Table S5. Diagnostic SNPs of each haplogroup of *O. edulis*

Position	Reference	haplogroup NS	haplogroup NEA	haplogroup WS	haplogroup SEA
24	G	.	A	A	A
77	A	.	G	G	G
98	T	.	C	C	C
197	C	.	T	T	T
204	A	.	G	G	G
213	G	.	A	A	A
219	C	.	T	T	T
253	G	.	.	.	A
258	G	.	A	A	A
339	C	.	T	T	T
361	T	.	C	C	C
480	C	.	T	T	T
544	G	.	A	A	A
665	G	.	.	.	A
667	A	.	G	G	G
791	T	.	C	C	C
797	C	.	T	T	T
806	G	.	A	A	A
809	A	.	G	G	G
815	A	.	G	G	G
872	A	.	C	C	C
914	A	.	.	.	G
987	A	.	G	G	G
1017	A	.	G	G	G
1096	T	.	C	C	C
1100	A	.	.	G	G
1129	A	.	G	G	G
1135	G	.	A	A	A
1156	T	.	.	C	.
1183	T	.	C	C	C
1213	C	.	.	T	.
1259	G	.	A	A	A

1267	A	.	G	G	G
1288	C	.	T	T	T
1446	A	.	G	G	G
1492	G	.	A	A	A
1508	G	.	.	A	.
1543	A	.	G	G	G
1555	A	.	G	G	G
1637	A	.	G	G	G
1700	A	.	G	G	G
1751	C	.	T	T	T
1756	A	.	G	.	.
1805	A	.	G	G	G
1814	T	.	C	C	C
1862	A	.	G	G	G
1865	A	.	G	G	G
1888	A	.	G	G	G
1910	A	.	G	G	G
1988	T	.	C	C	C
2017	T	.	C	C	C
2024	G	.	A	A	A
2027	A	.	G	G	G
2033	T	.	C	C	C
2036	A	.	G	G	G
2039	A	.	G	G	G
2093	C	.	T	T	T
2096	C	.	.	.	T
2210	A	.	G	G	G
2261	T	.	C	C	C
2312	G	.	.	A	A
2459	C	.	T	T	T
2473	A	.	G	G	G
2557	G	.	A	A	A
2564	G	.	.	A	.
2642	C	.	.	.	T

2657	G	.	.	T	.
2750	A	.	.	.	G
2819	C	.	T	T	T
2879	C	.	T	T	T
2900	A	.	G	G	G
2927	C	.	T	T	T
2987	T	.	.	.	C
2993	T	.	C	C	C
3035	G	.	.	A	.
3074	G	.	A	A	A
3152	G	.	.	A	.
3167	T	.	C	C	C
3182	C	.	.	T	.
3259	T	.	.	.	C
3264	C	.	T	T	T
3342	G	.	C	C	C
3363	C	.	T	T	T
3927	A	.	G	G	G
4026	T	.	.	C	C
4027	A	.	G	G	G
4031	T	.	.	C	C
4058	C	.	T	T	T
4111	G	.	.	.	A
4217	C	.	T	T	T
4243	G	.	A	A	A
4345	G	.	T	T	T
4352	G	.	A	A	A
4366	G	.	A	A	A
4384	T	.	C	C	C
4429	T	.	C	.	.
4557	G	.	.	.	A
4785	A	.	G	G	G
4892	C	.	T	T	.
4902	C	.	T	T	T

4906	A	.	G	G	G
4960	T	.	C	C	C
5090	G	.	A	A	A
5098	A	.	A	G	G
5108	G	.	A	A	A
5165	G	.	A	A	A
5174	A	.	.	G	.
5177	A	.	G	G	G
5219	A	.	G	G	G
5246	T	.	C	C	C
5375	T	.	.	.	C
5396	A	.	G	G	G
5419	C	.	.	.	T
5420	A	.	G	G	G
5423	T	.	C	C	C
5425	G	.	A	A	A
5489	T	.	C	C	C
5495	C	.	T	T	T
5615	A	.	.	.	G
5633	C	.	T	T	T
5650	A	.	G	G	G
5681	G	.	A	A	A
5729	A	.	G	G	G
5783	A	.	.	.	G
5816	T	.	C	C	C
5858	G	.	A	A	A
5885	G	.	.	A	.
5957	A	.	G	G	G
5981	C	.	T	T	T
5990	A	.	G	G	G
6044	A	.	G	G	G
6083	G	.	.	A	.
6152	C	.	T	T	T
6167	G	.	A	A	A

6173	C	.	T	T	T
6182	C	.	.	T	T
6185	G	.	.	.	A
6188	G	.	A	A	A
6379	T	.	C	C	C
6442	G	.	A	A	A
6458	C	.	T	T	T
6461	G	.	A	A	A
6464	T	.	C	C	C
6524	G	.	A	A	A
6593	A	.	T	T	T
6614	T	.	C	C	C
6634	T	.	C	C	C
6649	A	.	G	.	G
6758	A	.	G	G	G
6804	T	.	C	C	C
6879	G	.	A	A	A
6897	A	.	G	G	G
6996	T	.	C	C	.
7005	T	.	C	C	C
7032	A	.	G	G	G
7083	A	.	G	G	G
7130	G	.	A	A	A
7197	A	.	.	.	G
7200	C	.	.	T	.
7242	A	.	G	G	G
7449	A	.	G	G	G
7518	G	.	A	A	A
7528	G	.	A	A	A
7531	G	.	.	A	.
7545	G	.	A	A	A
7569	G	.	A	A	A
7597	C	.	T	T	T
7599	A	.	G	G	G

7603	C	.	T	T	T
7624	C	.	T	T	T
7654	A	.	G	.	.
7778	T	.	C	C	C
7840	C	.	T	T	T
7867	G	.	A	A	A
7954	C	.	T	T	T
8077	A	.	G	G	G
8094	C	.	.	.	T
8148	G	.	A	A	A
8158	G	.	A	A	A
8176	A	.	G	G	G
8293	C	.	T	T	T
8323	C	.	T	T	T
8377	T	.	C	C	C
8383	C	.	T	T	T
8395	G	.	.	A	.
8448	G	.	A	A	A
8449	A	.	G	G	G
8455	G	.	A	A	A
8467	G	.	A	A	A
8476	G	.	A	A	A
8491	T	.	C	C	C
8589	G	.	.	.	A
8680	T	.	.	C	C
8683	T	.	C	C	C
8841	A	.	G	G	G
8895	A	.	.	G	.
8989	C	.	T	T	T
8997	G	.	A	A	A
9022	G	.	A	A	A
9211	C	.	N	N	C
9217	T	.	C	C	C
9309	A	.	G	G	G

9416	T	.	.	C	.
9439	G	.	A	A	A
9449	A	.	G	G	G
9562	A	.	G	G	G
9591	T	.	C	G	C
9596	A	.	G	G	G
9607	G	.	A	A	A
9610	T	.	C	C	C
9639	A	.	G	G	G
9917	C	.	T	T	T
9940	T	.	C	C	C
9971	A	.	C	C	C
9977	A	.	G	G	G
10004	T	.	.	C	C
10025	A	.	G	.	.
10031	A	.	G	G	G
10049	G	.	A	A	A
10127	T	.	C	C	C
10130	G	.	.	A	.
10202	C	.	T	T	T
10277	C	.	.	T	.
10355	A	.	.	G	.
10424	T	.	.	C	.
10569	C	.	T	T	T
10629	C	.	T	T	T
10638	G	.	.	A	A
10701	A	.	T	T	T
10707	A	.	G	G	G
10749	C	.	T	T	T
10784	A	.	G	G	G
10785	G	.	A	A	A
10800	T	.	C	C	C
10830	G	.	A	A	A
10836	C	.	T	T	T

10881	A	.	G	G	G
10908	C	.	T	T	T
10923	G	.	A	A	A
10989	A	.	G	G	G
11016	A	.	G	G	G
11049	T	.	.	C	C
11073	A	.	G	G	G
11154	A	.	.	G	.
11183	T	.	C	C	C
11193	G	.	A	A	A
11232	C	.	C	C	C
11355	T	.	C	C	C
11382	T	.	C	C	C
11541	C	.	C	C	C
11552	G	.	A	A	A
11598	G	.	A	.	A
11650	T	.	C	C	C
11670	A	.	G	.	G
11739	C	.	T	T	T
11833	A	.	G	G	G
11897	A	.	G	G	G
11915	C	.	T	T	T
11999	A	.	G	G	G
12011	A	.	G	G	G
12020	A	.	G	G	G
12038	C	.	T	T	T
12071	T	.	C	C	C
12080	A	.	G	G	G
12161	T	.	C	C	C
12392	C	.	T	T	T
12422	A	.	G	G	G
12476	G	.	A	A	A
12494	G	.	A	A	A
12521	C	.	T	T	T

12533	T	.	.	C	.
12536	T	.	C	C	C
12548	G	.	A	A	A
12587	G	.	A	A	A
12665	A	.	G	G	G
12667	C	.	.	T	T
12687	T	.	C	C	C
12707	G	.	A	A	A
12734	C	.	.	C	.
12785	G	.	A	A	A
12810	T	.	N	N	N
12868	A	.	G	G	G
12912	C	.	T	T	T
12969	C	.	T	T	T
13002	G	.	A	A	A
13095	T	.	C	C	C
13098	A	.	G	G	G
13185	T	.	C	C	C
13194	C	.	T	T	T
13202	A	.	G	G	G
13221	G	.	.	.	A
13245	G	.	.	.	A
13287	C	.	.	.	T
13298	A	.	.	G	.
13368	G	.	.	A	.
13422	G	.	A	A	A
13427	G	.	A	A	A
13431	G	.	A	A	A
13437	G	.	A	A	A
13467	C	.	T	T	T
13497	G	.	A	A	A
13545	G	.	C	C	C
13554	A	.	G	G	G
13596	T	.	.	C	C

13662	T	.	C	C	C
13731	A	.	G	G	G
13737	C	.	A	A	A
13761	A	.	G	G	G
13776	A	.	G	G	G
13793	A	.	G	G	G
13794	G	.	A	A	A
13806	T	.	C	C	C
13860	A	.	G	G	G
13877	A	.	G	.	.
13878	T	.	C	C	C
13889	G	.	A	A	A
13946	G	.	.	.	A
14054	G	.	.	A	A
14073	G	.	A	A	A
14097	A	.	G	G	G
14184	A	.	G	G	G
14226	C	.	T	T	T
14240	A	.	G	G	G
14247	A	.	.	.	G
14310	A	.	G	G	G
14361	T	.	.	C	.
14575	T	.	.	C	.
14616	A	.	G	G	G
14631	A	.	T	T	T
14636	A	.	G	G	G
14682	G	.	C	C	C
14692	A	.	G	G	G
14706	A	.	G	G	G
14710	T	.	.	.	C
14725	A	.	G	G	G
14729	A	.	G	G	G
14752	T	.	C	C	C
14806	G	.	A	A	A

14812	T	.	C	C	C
14825	A	.	G	G	G
14839	G	.	A	A	A
14850	A	.	G	G	G
14879	A	.	N	N	N
14898	A	.	G	G	G
14899	T	.	G	G	C
14908	A	.	G	G	G
14959	G	.	A	A	A
14991	C	.	.	T	T
15023	A	.	G	G	G
15025	A	.	G	G	G
15117	T	.	.	C	.
15171	A	.	G	G	G
15182	A	.	G	G	G
15188	A	.	C	C	C
15195	A	.	G	G	G
15312	T	.	C	C	C
15385	A	.	.	.	G
15412	A	.	.	G	G
15450	G	.	.	A	.
15452	T	.	C	C	C
15458	T	.	C	C	C
15482	C	.	.	T	T
15484	G	.	A	A	A
15497	A	.	G	G	G
15533	T	.	C	C	C
15569	G	.	A	A	.
15688	G	.	A	A	A
15695	C	.	G	G	G
15803	G	.	A	A	A
15813	A	.	G	G	G
15927	C	.	T	T	T
15951	T	.	.	C	C

15981	A	.	G	G	G
15987	T	.	C	C	C
16050	C	.	T	T	T
16056	T	.	C	C	C
16089	T	.	C	C	C
16137	G	.	A	A	A
16139	A	.	G	.	.
16194	A	.	G	G	G
16196	T	.	.	C	.
16290	A	.	T	T	T
16293	G	.	.	.	A
16341	T	.	C	C	C
16344	G	.	T	T	T
